# Predictive Value of Magnetic Resonance Imaging in Risk Stratification and Molecular Classification of Endometrial Cancer

**DOI:** 10.3390/cancers16050921

**Published:** 2024-02-25

**Authors:** Hanna Bae, Sung Eun Rha, Hokun Kim, Jun Kang, Yu Ri Shin

**Affiliations:** 1 Department of Radiology, Seoul St. Mary’s Hospital, College of Medicine, The Catholic University of Korea, Seoul 14662, Republic of Korea; qogkssk416@naver.com (H.B.); serha@catholic.ac.kr (S.E.R.); walehn@gmail.com (H.K.); 2 Department of Hospital Pathology, Seoul St. Mary’s Hospital, College of Medicine, The Catholic University of Korea, Seoul 14662, Republic of Korea; jkang.alien@gmail.com

**Keywords:** endometrial cancer, magnetic resonance imaging (MRI), risk stratification, molecular classification, p53 status, microsatellite instability

## Abstract

**Simple Summary:**

This study investigated the ability of MRI to assist in predicting different risks and genetic types of endometrial cancer. The goal was to determine whether MRI could be used to predict the aggressiveness of cancer and identify its genetic characteristics, thereby guiding doctors in choosing the best treatment options. We found that women at higher risk typically had larger tumors and more impeded water diffusion than low-risk patients did. We also attempted to connect MRI data with the genetic profile of the cancer patients. Although the results were obtained early, they suggested that MRI could help identify the genetic features of tumors, which is important for customizing treatment for each patient. Our study adds to the evidence that MRI may be a key tool not only for diagnosing endometrial cancer but also for planning more personalized treatments for patients based on the specific traits of their cancers observed via MRI scans.

**Abstract:**

This study evaluated the magnetic resonance imaging (MRI) findings of endometrial cancer (EC) patients and identified differences based on risk group and molecular classification. The study involved a total of 175 EC patients. The MRI data were retrospectively reviewed and compared based on the risk of recurrence. Additionally, the associations between imaging phenotypes and genomic signatures were assessed. The low-risk and non-low-risk groups (intermediate, high-intermediate, high, metastatic) showed significant differences in tumor diameter (*p* < 0.001), signal intensity and heterogeneity on diffusion-weighted imaging (DWI) (*p* = 0.003), deep myometrial invasion (involvement of more than 50% of the myometrium), cervical invasion (*p* < 0.001), extrauterine extension (*p* = 0.002), and lymphadenopathy (*p* = 0.003). Greater diffusion restriction and more heterogeneity on DWI were exhibited in the non-low-risk group than in the low-risk group. Deep myometrial invasion, cervical invasion, extrauterine extension, lymphadenopathy, recurrence, and stage discrepancy were more common in the non-low-risk group (*p* < 0.001). A significant difference in microsatellite stability status was observed in the heterogeneity of the contrast-enhanced T1-weighted images (*p* = 0.027). However, no significant differences were found in MRI parameters related to TP53 mutation. MRI features can be valuable predictors for differentiating risk groups in patients with EC. However, further investigations are needed to explore the imaging markers based on molecular classification.

## 1. Introduction

Endometrial cancer was the sixth most common tumor among female individuals and the fourth leading cause of death due to gynecological cancer among women worldwide in 2018 [1]. The incidence and prevalence of endometrial cancer are progressively increasing [2]. Risk factors for endometrial cancer include obesity, hypertension, hyperinsulinemia, and prolonged exposure to unopposed estrogen [3,4]. Endometrial cancers are traditionally divided into two main subtypes based on their histological features, clinical behavior, and epidemiology. Type I tumors, primarily consisting of grade 1 or 2 endometrioid carcinomas, have a favorable prognosis due to their estrogen responsiveness. In contrast, type II cancers encompass aggressive histological types, such as grade 3 endometrioid carcinomas, serous carcinoma, clear-cell carcinoma, mixed carcinoma, undifferentiated carcinoma, carcinosarcoma, mesonephric-like, and gastrointestinal-type mucinous carcinomas, all of which carry a heightened risk of relapse [5,6,7]. Current guidelines, including those from the European Society for Medical Oncology (ESMO), identify several prognostic factors, such as histological subtype, high-grade cells, deep myometrial invasion, lymphovascular space invasion, lymph node metastasis, and tumor size [8]. Preoperatively identifying high-risk factors in patients can significantly impact therapeutic management by preventing over- and undertreatment and extending survival.

The advent of molecular profiling in oncology has heralded a notable shift from purely morphological to molecular-based classification of endometrial cancer. The classification of endometrial cancer, as initially introduced by The Cancer Genome Atlas (TCGA) initiative, effectively addresses this need based on both somatic mutational load and somatic copy-number variations. The TCGA studies have identified four molecular subgroups characterized by POLE mutation, mismatch-repair deficiency, TP53 mutation, and low copy number without a specific driver mutation, each with a distinct prognosis: the POLE/ultramutated subtype with favorable outcomes; the microsatellite instability-high subtype, typically endometrioid or undifferentiated carcinomas with intermediate prognosis; the somatic copy number-high subtype, including serous and high-grade endometrioid cancers, associated with poorer outcomes; and the somatic copy number-low subtype, involving endometrioid and clear-cell carcinomas, where the prognosis is influenced by estrogen receptor status and histological grading [9,10,11,12,13,14]. Its molecular classification is likely the most innovative progress in the endometrial carcinoma field in recent years. Preoperative molecular assessment is not only valuable for risk stratification, but it can also be utilized to make tailored surgical decisions, determine the necessity of adjuvant therapy, and design individualized follow-up plans [15]. This has led to the integration of molecular criteria into the revised International Federation of Gynecology and Obstetrics (FIGO) staging of endometrial cancer in 2023, emphasizing the importance of molecular characteristics in determining prognosis and treatment strategies [16].

MRI is recognized for its critical role in the preoperative evaluation of endometrial cancer, as it offers detailed insights into disease extent and assists in risk stratification [17]. Recent guidelines for the management of patients diagnosed with endometrial cancer, as outlined by Restaino et al., have begun to incorporate imaging management, particularly focusing on the role of MRI in conjunction with molecular classification [18]. This integration of imaging into guidelines aligns with recent advances in molecular classification and the increasing emphasis on precision oncology. However, despite the acknowledged utility of MRI for informing treatment plans, especially for avoiding unnecessary procedures in low-risk patients, the correlation between MRI features and risk stratification remains underexplored. Despite its low spatial resolution, DWI can be used to effectively identify tumors with restricted diffusion, reflecting tissue cellularity and membrane integrity. This capability not only enhances DWI’s role in staging endometrial cancer, particularly in determining myometrial invasion, but also suggests its emerging utility in differentiating risk categories [19,20,21]. Although few recent radiomics models could be useful for preoperative endometrial cancer risk stratification, the application of these models in clinical practice is limited due to the limited availability of computerized instruments [22,23,24]. Endometrial cancer often harbors mutated TP53, which promotes blood-vessel growth and might influence the response to antiangiogenic drugs such as bevacizumab. Imaging biomarkers for detecting TP53 could guide treatment but are still under development. Furthermore, preoperative detection of molecular classification can provide a basis for personalized and precise treatment of endometrial cancer. Despite the recognition of the detailed preoperative assessment capabilities of MRI for endometrial cancer, its full potential in conjunction with molecular classification has not been fully explored. Therefore, in our study, we aimed to evaluate the MRI findings of patients with endometrial cancer according to the risk group and clarify the imaging characteristics of the molecular subtypes.

## 2. Materials and Methods

### 2.1. Study Population

**Figure 1:**
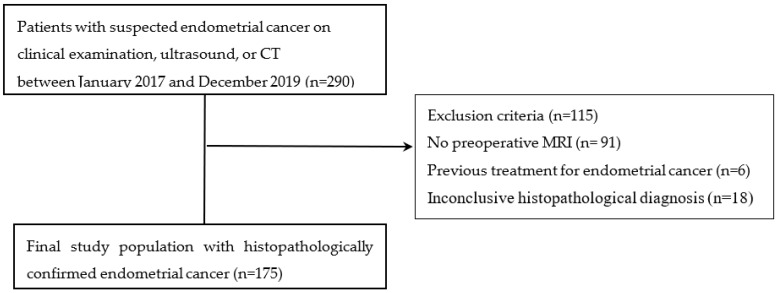
Flow diagram illustrating the inclusion of study participants.

The research was authorized by the relevant Institutional Review Board, and obtaining informed consent was waived due to the retrospective nature of the study design. After reviewing clinical and pathological findings from our institutional database, 290 patients with suspected endometrial cancer were identified between January 2017 and December 2019. The inclusion criteria were pathologically confirmed endometrial cancer patients, a pelvic MRI examination performed within 2 weeks before the operation, and no other treatment received before the examination. Patients without preoperative MRI data (*n* = 91), previous treatment for endometrial cancer (*n* = 6), or inconclusive histopathologic diagnoses (*n* = 18) were excluded from this study. Ultimately, 175 patients were enrolled in this study (Figure 1). Patients were first stratified according to the risk of recurrence by the ESMO–ESGO–ESTRO Consensus Conference on Endometrial Cancer and then categorized into two groups [25]. Low-risk patients were classified into the low-risk group, while intermediate-risk, high-intermediate-risk, high-risk, advanced-risk, and metastatic-risk patients were classified into the non-low-risk group.

### 2.2. Imaging Techniques

All MRI scans were carried out using a 3.0-T scanner (Verio, Siemens Healthcare, Erlangen, Germany) equipped with a pelvic phased-array coil. The patients were instructed to fast for 3 h prior to the scan and were administered an antispasmodic medication (Buscopan, Boehringer Ingelheim, Ingelheim, Germany) intravenously just before the imaging process commenced. This was done to minimize bowel peristalsis. The MRI protocol consisted of turbo spin–echo T2-weighted sequences in the sagittal, axial, and coronal planes using radial blades (BLADE: TR/TE = 4000 ms/118 ms; slice thickness = 6 mm; flip angle = 138°; matrix = 320 × 320; FOV = 240 × 240 mm). Axial T1-weighted sequences were acquired both with and without fat suppression (TR/TE = 650 ms/12 ms; slice thickness = 6 mm; flip angle = 150°; matrix = 320 × 224; FOV = 240 × 240 mm). Diffusion-weighted imaging (DWI) sequences were obtained using axial and sagittal single-shot isotropic echo-planar sequences with fat saturation. Free-breathing DWI was performed with b-values of 0, 50, and 1000 s/mm^2^ (TR/TE = 6200 ms/80 ms; slice thickness = 6 mm; flip angle = 90°; matrix = 100 × 100; FOV = 240 × 240 mm). The scanner automatically generated an apparent diffusion coefficient (ADC) map. Subsequently, a dynamic multiphase contrast-enhanced MRI was performed by using a three-dimensional gradient echo T1-weighted sequence after the administration of 0.2 mmol of gadolinium per kg of body weight at a rate of 1.0 mL/s gadoteridol (ProHance, Bracco, Milan, Italy). Dynamic MR imaging was performed every 30 sec for 4.5 min without breath-holding in the sagittal plane. After dynamic MR imaging, contrast-enhanced T1-weighted spin–echo MR imaging was performed 5 min after administration of the contrast material in the axial and sagittal planes with the same parameters as those of unenhanced T1-weighted spin–echo imaging (Table 1).

### 2.3. Image Analysis and Interpretation

Two radiologists with 20 years and 4 years of experience in gynecological imaging individually reviewed all the MR images. The reviewers were aware that all patients had endometrial cancer, but they were blinded to the clinical information, histopathological examination results, and follow-up. The reviewers assessed various aspects of the tumor presentation and characteristics, including size, growth pattern, degree of signal intensity (SI), and heterogeneity of endometrial cancer. The maximum tumor diameter was measured where it appeared largest on the three planes of the T2-weighted images. The tumor-growth patterns were categorized as either infiltrative or expansile. An infiltrative pattern was characterized by an indistinct and irregular boundary between the tumor and myometrium or adjacent tissues. In contrast, the expansile pattern demonstrated a distinct and smooth demarcation between these structures. The SI and heterogeneity of endometrial cancer were assessed on T2-, diffusion-, and fat-suppressed contrast-enhanced T1-weighted images. The degree of SI (hypointensity, isointensity, and hyperintensity) was compared with that of the normal myometrium. Restricted water diffusion was identified by the presence of increased signal intensity on DWI with b values of 1000 s/mm^2^ coupled with reduced values on corresponding ADC maps. Using the region of interest (ROI), the apparent diffusion coefficient (ADC) values of the tumors were measured. All ROIs were precisely positioned on the solid components of the tumors, excluding the necrotic area in the slice with the maximum diameter possible. Additionally, the following were evaluated: deep myometrial invasion, cervical stromal invasion, extrauterine extension, rectal or bladder invasion, ascites, peritoneal dissemination, the presence of metastatic lymph nodes, and distant metastases. Deep myometrial invasion was demonstrated as a tumor involving greater than 50% of the myometrium. On dynamic multiphase contrast-enhanced MRI, deep myometrial invasion was evaluated in the equilibrium phase (2–3 min postinjection) because the tumor-to-myometrial contrast ratio was the highest at that point [26,27]. Cervical stromal invasion was evaluated in the delayed phase (4–5 min postinjection) [28]. Extrauterine extension was defined as disruption of the hypointense signal of the uterine serosa on T2-weighted images with irregularity of the outer smooth uterine contour and loss of the normal rim enhancement of the outer myometrium on contrast-enhanced images. Lymphadenopathy was defined as a node occurring when it was more than 8 mm in short-axis diameter [29].

The group with recurrence was defined as patients who had undergone a biopsy to confirm recurrence because of abnormal focal findings on postoperative computed tomography (CT) or MRI. Patients without recurrence were defined as patients who had pathologically or clinically confirmed benign lesions that had appeared as focal abnormal findings on postoperative CT or MRI. Table 2 shows the Cohen’s kappa values for the assessment of MRI parameters by the two reviewers. Kappa values ranging from 0.66 to 1.00 indicate a consistently high level of agreement between the reviewers.

### 2.4. Histopathological Analysis

All lesion specimens were obtained surgically and were processed by an experienced pathologist. The histological subtype, grade, and lymphovascular space invasion were confirmed by hematoxylin–eosin staining. The staging was carried out according to the 2018 version of the FIGO staging standard. The TP53 status was evaluated by immunohistochemical staining, where nonstaining was considered the wild-type group, and faint, moderate, and strong staining were considered the mutant group. The microsatellite instability (MSI) assay was carried out using DNA extracted from flash-frozen tumors from hysterectomy specimens. Mutational analysis was performed using the U-TOP MSI Detection Kit Plus (Seasun Biomaterials, Daejeon, Republic of Korea). This kit, which is based on a peptide nucleic acid (PNA)-mediated real-time polymerase chain reaction (PCR) approach, determines the MSI status by analyzing amplicon melting of five quasimonomorphic mononucleotide repeat markers (NR21, NR24, NR27, BAT25, and BAT26) and an internal control. Samples with alterations in more than one MSI marker are classified as MSI-high, while those with a single alteration or no alteration are classified as MSI-low or microsatellite stable (MSS), respectively. In a previous study, no significant clinicopathological or molecular differences were found between MSI-low and MSS colorectal cancers, so they were combined for statistical analysis [30]. MSI testing, originally for colorectal cancer, is applicable in endometrial cancer due to shared DNA mismatch-repair defects, a common aspect in several cancers [31,32,33,34]. Ultimately, risk stratification was evaluated in all 175 patients, TP53 status was evaluated in 86 patients, and MSI status was evaluated in 36 patients.

### 2.5. Statistical Analysis

In cases where different results were obtained, the reviewers discussed and reached a conclusion by consensus. The results achieved by consensus were used for the statistical analyses, except for interobserver agreement. Associations between MRI parameters and risk group and between MRI parameters and immunohistologic molecular expression were assessed using appropriate statistical tests. Chi-squared and Fisher’s exact tests were employed for categorical data analysis, while Wilcoxon rank-sum and Kruskal–Wallis tests were utilized for continuous data, depending on the data distribution and sample size. The interobserver variation between the two observers was determined using Cohen’s kappa value, calculated using the R program (v4.0.2) and explicitly stated as the kappa value. The statistical analyses were conducted using SAS version 9.4 (SAS Institute, Cary, NC, USA). *p* < 0.05 indicated statistical significance, and pairwise deletion was used to handle missing data.

## 3. Results

The characteristics of the patients are summarized in Table 3. The mean age was 55.12 (27–84) years, and the FIGO stage IA was the most prevalent stage (*n* = 111, 63.43%). In this study, 175 patients, including 150 patients with endometrioid adenocarcinoma, 3 with mucinous carcinoma, 8 with serous carcinoma, 4 with clear-cell carcinoma, and 10 with carcinosarcoma, were enrolled. The mean follow-up period was 44.4 months (range: 36.1–72.5 months).

**Figure 2:**
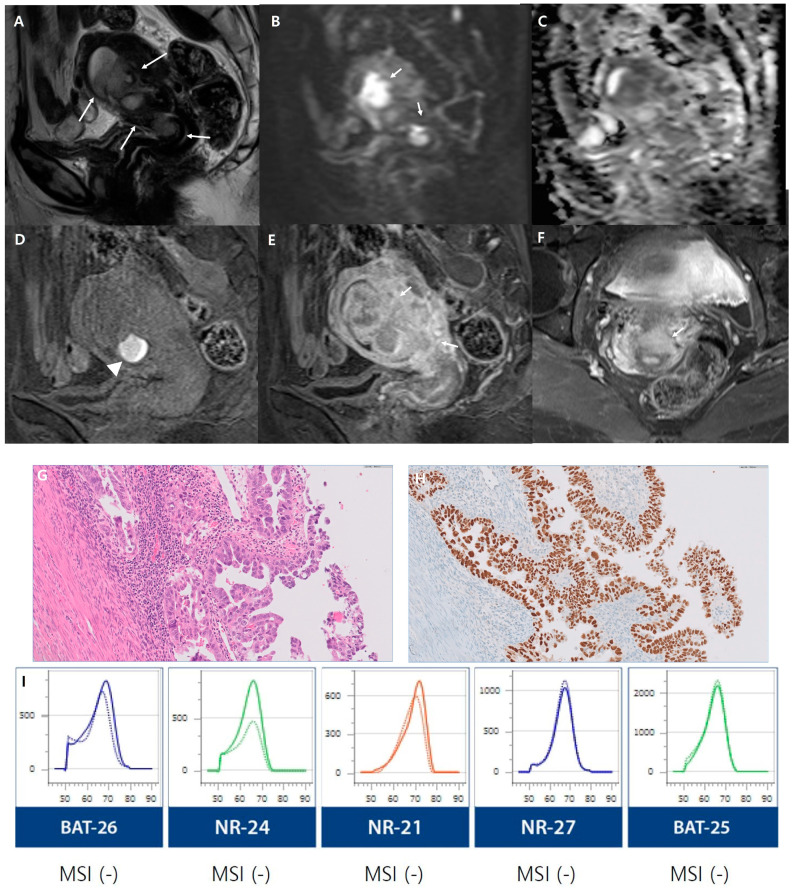
A 56-year-old woman with non-low-risk endometrial cancer (high-grade serous carcinoma, lymphovascular space invasion). (**A**) A sagittal T2-weighted image depicts a large, ill-demarcated intrauterine mass exhibiting invasive growth (arrows), which appears with predominantly heterogeneous intermediate signal intensity. On the diffusion-weighted image acquired at b = 1000 s/mm^2^ (**B**), the mass is highlighted by heterogeneous high signal intensity (arrows), while it demonstrates hypointensity on the apparent diffusion coefficient (ADC) map (**C**), suggestive of restricted diffusion. (**D**,**E**) Dynamic contrast-enhanced (DCE) MRI sequences reveal a region within the mass of high signal intensity on precontrast T1-weighted images, indicative of hemorrhage (arrowhead in (**D**)). The mass, presenting with deep myometrial invasion, exhibits heterogeneous enhancement compared to the surrounding myometrium during the equilibrium phase (2–3 min postcontrast administration, arrows in (**E**)). (**F**) An axial delayed phase image shows evidence of cervical stromal invasion (arrow). (**G**) Histopathological examination (hematoxylin and eosin stain, magnification ×400) reveals disordered glandular growth with pale, enlarged nuclei and prominent nucleoli. (**H**) p53 immunohistochemistry (magnification ×400) indicates diffuse and intense nuclear staining within the tumor tissue, consistent with TP53 mutation. (**I**) Microsatellite instability analysis for five markers, using PNA probe-mediated real-time PCR sensing for the detection of MSI status. This method reveals stability at microsatellite loci, indicating microsatellite stable (MSS) status, as the melting peaks between tumor DNA (dashed lines) and normal DNA (reference lines) do not differ by more than 3 °C.

**Figure 3:**
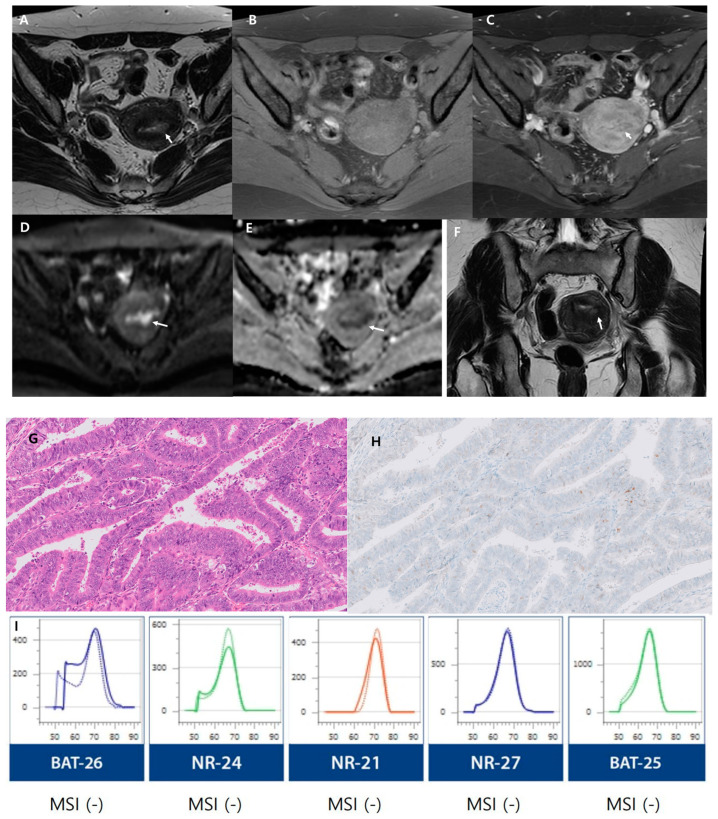
A 52-year-old woman with low-risk endometrial cancer (endometrioid carcinoma, FIGO grade 2). (**A**,**F**) Axial and coronal T2-weighted images show a 1.5 cm, ill-defined mass in the uterine fundus (arrows) with heterogeneous intermediate signal intensity. (**B**,**C**) Axial fat-saturated contrast-enhanced T1-weighted image reveals heterogeneous enhancement without deep myometrial invasion. (**D**) Diffusion-weighted image (b = 1000 s/mm^2^) highlights the mass with heterogeneous high signal intensity (arrow), while the apparent diffusion coefficient (ADC) map (**E**) shows hypointensity, suggesting restricted diffusion. (**G**) Histopathological examination (hematoxylin and eosin stain, magnification ×400) reveals well-differentiated glandular architecture with columnar epithelial cells showing mild-to-moderate nuclear atypia. (**H**) p53 immunohistochemistry (magnification ×400) demonstrates absent nuclear positivity in tumor cells, consistent with wild-type TP53. (**I**) Microsatellite instability analysis for five markers, using PNA probe-mediated real-time PCR sensing for the detection of MSI status. This method reveals stability at microsatellite loci, indicating microsatellite stable (MSS) status, as the melting peaks between tumor DNA (dashed lines) and normal DNA (reference lines) do not differ by more than 3 °C.

We conducted assessments based on risk groups as outlined by the European Society for Medical Oncology (ESMO) clinical practice guidelines [25]. A comparison of MRI parameters between the low-risk and non-low-risk groups is presented in Table 4. The low-risk and non-low-risk groups showed significant differences in the maximum tumor diameter (*p* < 0.001), SI on DWI (*p* = 0.003), heterogeneous SI on DWI (*p* = 0.003), deep myometrial invasion, cervical stromal invasion (*p* < 0.001), extrauterine extension (*p* = 0.002), and lymphadenopathy (*p* = 0.003). Greater impeded water diffusion and more heterogeneous SI on DWI were exhibited in the non-low-risk group than in the low-risk group. In addition, deep myometrial invasion, cervical stromal invasion, extrauterine extension, and lymphadenopathy were more common in the non-low-risk group than in the low-risk group (Figure 2 and Figure 3).

We examined recurrence outcomes within these defined risk categories and assessed the concordance between the FIGO stage and clinical stage. Table 5 shows the association between tumor recurrence and stage discrepancy according to the risk group. The non-low-risk group displayed a greater incidence of tumor recurrence and stage discrepancy than did the low-risk group (*p* < 0.001).

In addition, among patients with pathologically confirmed molecular subtypes, a comparative analysis of MRI findings was conducted, stratified by pathologic results indicating the TP53 and MSI status. Table 6 and Table 7 show the imaging value of the molecular subtypes according to the presence of TP53 mutations and MSI status. Regarding TP53 status, 24 patients were in the TP53-mutant group, and 62 patients were in the TP53-wild-type group. MRI parameters exhibited no statistically significant correlation with the presence of TP53 mutations (*p* > 0.05). Regarding the MSI status, 36 patients had pathological results indicating MSI status, with 8 patients classified as the MSI group and 28 patients as the MSS group. A significant difference was observed in the heterogeneous SI of the tumor on contrast-enhanced T1-weighted images (*p* = 0.027). The MSS group showed more heterogeneous enhancement than the MSI group.

## 4. Discussion

With our study, we have made considerable strides in distinguishing between non-low-risk and low-risk endometrial cancer groups through MRI, revealing that non-low-risk patients have larger tumors, more restricted water diffusion, and increased heterogeneity in signal intensity on DWI. These patients also had a greater frequency of invasive characteristics, such as deep myometrial invasion, cervical stromal invasion, extrauterine extension, and lymphadenopathy that correlated with a greater rate of tumor recurrence and stage discrepancy. Our exploration of imaging characteristics according to molecular classification is preliminary and has not yet yielded definitive results.

We found that the maximum tumor diameter significantly differed between risk groups, echoing previous findings that larger tumor sizes are associated with poorer prognoses [35,36]. The invasive features prevalent in the non-low-risk groups align with the findings of other studies, such as those of Maria Ali et al. and Stephanie Nougaret et al., which linked tumor size to lymph node metastasis and myometrial invasion depth, respectively [37,38]. These aggressive tumor characteristics are indicative of a higher risk category and suggest a propensity for more extensive disease.

In this study, we opted to stratify risk using the ESMO guidelines because of the synthesis of emerging molecular markers with conventional risk factors. However, we recognize the importance of considering alignment with other major guidelines, such as the Gynecologic Oncology Group (GOG)/International Federation of Gynecology and Obstetrics (FIGO) and the National Comprehensive Cancer Network (NCCN), which can differ in their risk criteria and recommendations [18]. An accurate assessment of parameters, such as tumor size and myometrial invasion, provides vital data to inform guideline-based therapy across standards. For instance, in discordant scenarios, MRI findings can support personalized decisions regarding radiation therapy versus systemic therapy [39]. Future research should focus on integrating MRI data with multidisciplinary inputs and evolving guidelines to enhance personalized endometrial cancer care [40].

MRI provides vital adjunctive data to determine the extent of surgery, as does histopathology. When MRI reveals low-risk features, such as tumor size, limited myometrial invasion, no diffusion restriction, and the absence of suspicious lymph nodes, it may guide less radical resection for early tumors to minimize morbidity. However, MRI cannot definitively exclude high-risk diseases that warrant comprehensive surgery. For advanced MRI features, such as larger size, deep myometrial invasion, restricted diffusion suggestive of aggressive cellular behavior, and suspicious lymph nodes, more extensive surgery is necessary. Additionally, in qualified early-stage patients, MRI facilitates fertility preservation by facilitating patient selection for conservative approaches when performed alongside histopathological criteria [18]. Ultimately, MRI provides complementary prognostic insights into tumor extent and risk profile to complement tissue diagnosis and aid surgical planning but does not supersede pathological staging.

Radiomics has gained significant recognition as a novel approach for obtaining quantitative data on the microscopic features of tissues from clinical images, and it has emerged as a critical component in scientific research in the recent past. In comparison to other radiomics studies, our study stands out due to its applicability within a clinical setting without the need for additional specialized equipment or training. Our study leverages standard MRI sequences that are already a mainstay in clinical practice, thus providing immediate, actionable insights that can be seamlessly integrated into existing diagnostic pathways.

Although direct histopathological analysis remains the gold standard, preoperative prediction of molecular subtypes using MRI could have significant clinical implications. For example, identifying high-risk molecular features via MRI could inform the decision for more extensive surgical procedures, such as pelvic lymphadenectomy or neoadjuvant therapy, to improve outcomes. Conversely, for low-risk patients identified through MRI, less invasive surgical approaches, such as laparoscopic hysterectomy, could be considered, reducing the surgical burden and potential complications. Furthermore, MRI could offer a noninvasive and potentially cost-effective alternative to routine molecular characterization, particularly in resource-constrained settings. For instance, in patients in which MSI testing is not readily available, MRI-based assessment could provide a preliminary indication of tumor aggressiveness, guide initial treatment decisions, and potentially avoid unnecessary molecular testing. This could be especially beneficial for patients who are unable to undergo immediate surgery because of medical comorbidities or for whom surgery poses significant risks. Like the ROME study, which aimed to improve disease characterization and reduce costs through radiomic analysis of ultrasound images, we believe that MRI can play a complementary role in the overall risk stratification and management of endometrial cancer patients [41]. By providing preoperative insights into molecular subtypes, MRI can potentially optimize treatment strategies, minimize unnecessary procedures, and improve patient outcomes. We are confident that future research, including larger studies such as ROME, will further validate and refine the use of MRI in this context.

In clinical practice, molecular and genetic profiling traditionally depend on tissue obtained from surgical resection or biopsy. However, noninvasive imaging, through the identification of imaging markers correlating with genetic profiles, offers a promising frontier for tumor characterization. Although the characteristic imaging markers of each subtype are useful for predicting patient prognosis and improving treatment strategies, our study revealed no significant differences in MRI parameters related to TP53 mutation. The TP53 gene mutation can promote the expression of vascular endothelial growth factor (VEGF), which can increase the permeability of blood vessels while inducing the growth of tumor blood vessels [42]. However, our study showed no significant association between TP53 mutation and MRI-detectable changes in angiogenesis. This might indicate a lack of sensitivity in MRI to capture these subtle phenotypic changes or the influence of other overriding factors that mask the effects of TP53 mutation.

TP53 is a critical tumor suppressor that regulates cell-cycle arrest, apoptosis, DNA repair, and angiogenesis. Mutations in TP53 lead to the loss of these regulatory functions and are detected in 20–30% of endometrial cancers; these mutations are associated with aggressive disease features, including high-grade, advanced stage, lymphovascular invasion, nonendometrioid histology, and poor prognosis [43]. TP53 dysfunction also induces vascular endothelial growth factor (VEGF) expression and angiogenesis. As VEGF inhibitors such as bevacizumab are emerging as treatments for endometrial cancer, detecting the TP53 status could inform antiangiogenic therapy decisions [44]. Preoperative identification of TP53 mutations may guide surgical planning and adjuvant therapy selection. For patients with mutant TP53 whose tumors exhibit elevated VEGF, antiangiogenic therapy with bevacizumab may hold particular promise [45]. However, the use of imaging biomarkers for predicting TP53 mutations is still in its infancy, and further development and validation are needed [46,47]. 

MSI-high endometrial cancer patients commonly exhibit a higher tumor grade, deeper myometrium invasion, and a greater incidence of lymph node metastasis. Ahmed et al. reported that MSI was associated with low signal intensity on late T1W DCE MRI [48]. However, these correlations were not observed in this study. While our single finding on heterogeneous SI on contrast-enhanced T1-weighted images in the context of MSI suggests a potential imaging biomarker, larger studies are necessary for validation. Furthermore, advancements in MRI technology, such as improved pulse sequences and reconstruction algorithms, hold promise for augmenting the quality of both anatomic and functional imaging in endometrial cancer [49,50,51]. These innovations may ultimately enhance our ability to noninvasively discern molecular subtypes, supporting a more nuanced approach to personalized treatment planning.

Our study highlights the potential of MRI for risk stratification beyond histopathology, paving the way for future research to solidify its prognostic value and improve clinical outcomes for patients with endometrial cancer. Incorporating estrogen-related factors would add another layer of depth, but additional data collection and potentially a prospective study design would be needed for accurate analysis of the influence of these factors on both MRI features and molecular subtypes. Given the scope and resources of our current study, we were unable to fully segregate patient data based on estrogen levels or related clinical factors. We remain committed to exploring this avenue through existing data analysis, pilot studies, and collaborations, aiming to elucidate the relationships between estrogen levels, MRI features, and molecular classification, ultimately leading to personalized treatment strategies.

Despite these insightful findings, our study has several limitations that need to be acknowledged. First, this was a retrospective, single-institution study with a limited sample size. A larger-scale study is needed to confirm these findings. Second, not all patients underwent both diffusion-weighted and contrast-enhanced MRI, which limits the comprehensiveness of our data. This disparity might have masked some potential correlations or trends. Third, a relatively small number of patients underwent immunohistochemical staining and PCR testing, although this study obtained the largest number of molecular classification results among the radiology studies. The absence of molecular classification results for all endometrial cancer patients restricts our ability to fully understand the relationship between genomic profiles and imaging findings. Fourth, immunohistochemistry (IHC) was used to detect the presence and overexpression of the TP53 protein in surgical resection specimens, as this protein is a surrogate marker for TP53 gene mutations. We acknowledge that this approach has limitations compared to direct TP53 gene sequencing, which can lead to definitive mutation identification. However, IHC is considered a reliable and feasible method within the context of clinical practice. We agree that the optimal TP53 analysis methodology is an important consideration, especially given the potential implications of TP53 status for angiogenesis and antiangiogenic therapy decisions. Further studies utilizing advanced genomic techniques, such as next-generation sequencing or HRD analysis, can provide valuable insights in this regard. Finally, manual ROI segmentation introduces potential observer bias, pointing to the need for more sophisticated image-analysis techniques, such as automatic/semiautomatic segmentation.

## 5. Conclusions

In conclusion, our study has shown the relative strength of MRI in the risk stratification of endometrial cancer, particularly through DWI. Considering the increasing impact of molecular analysis on the future of personalized treatment strategies in endometrial cancer care, our findings indicate that further research is warranted to elucidate the connections between MR imaging characteristics and the molecular subtypes of endometrial cancer.

## Figures and Tables

**Table 1:** Contrast-enhancement process.

Step	Procedure	Details	Scan Time	Phase
1	Patient Preparation	-Fasting for 3 h; -Administration of antispasmodic drug intravenously.	NA	NA
2	Baseline Scans	-Conducting initial MRI scans before contrast injection.	NA	NA
3	Contrast Administration	-Administering 0.2 mmol/kg gadoteridol; -Automated power injector at a rate of 1.0 mL/s followed by a flush within 30 mL of 0.9% sterile saline.	NA	NA
4	Multiphase 3D fat-saturated contrast-enhanced T1-weighted imaging	-Performing imaging every 30 sec for 4.5 min in the sagittal plane without breath-holding.	0–30 s 60–90 s 120–150 s 180–210 s 240–270 s	Early phase (Subendometrial enhancement assessment) Equilibrium phase (Maximal tumor-to-myometrium contrast) Delayed phase (Cervical stromal invasion assessment)
5	Postcontrast T1-weighted imaging	-Conducting scans 5 min after contrast material administration in axial and sagittal planes with parameters similar to unenhanced T1-weighted imaging.	NA	NA

NA = not applicable.

**Table 2:** Kappa statistics of interobserver agreement.

Imaging Factor	Kappa (95% Confidence Interval)
Growth pattern	0.81 (0.72, 0.9)
SI on T2WI	0.74 (0.63, 0.86)
Heterogeneous SI on T2WI	0.91 (0.84, 0.99)
SI on CET1	0.86 (0.74, 0.98)
Heterogeneous SI on CET1	0.92 (0.86, 0.99)
SI on DWI	0.90 (0.81, 0.99)
Heterogeneous SI on DWI	0.95 (0.88, 1.00)
Deep myometrial invasion	0.94 (0.88, 1.00)
Cervical stromal involvement	0.97 (0.91, 1.00)
Extrauterine extension	1.00 (1.00, 1.00)
Rectal or bladder invasion	1.00 (1.00, 1.00)
Abnormal ascites	0.66 (0.05, 1.00)
Peritoneal dissemination	1.00 (1.00, 1.00)
Lymphadenopathy	0.83 (0.65, 1.00)

SI = signal intensity; T2WI = T2-weighted image; CET1 = contrast-enhanced T1-weighted image; DWI = diffusion-weighted image.

**Table 3:** Patients’ characteristics.

Age, mean (range)	55.12 (27–84)
Postmenopausal, *n* (%)	108 (61.7%)
FIGO Stage (2018), *n* (%)	
I	131 (74.86)
IA	111 (63.43)
IB	20 (11.43)
II	11 (6.29)
III	24 (13.71)
IIIA	7 (4)
IIIB	2 (1.14)
IIIC1	5 (2.86)
IIIC2	10 (5.71)
IV	9 (5.14)
IVA	0 (0)
IVB	9 (5.14)
Endometrial cancer subtype, *n* (%)	
Endometrioid adenocarcinoma	150 (85.71)
Grade 1	81 (46.29)
Grade 2	48 (27.43)
Grade 3	21 (12.00)
Mucinous carcinoma	3 (1.71)
Serous carcinoma	8 (4.57)
Clear-cell carcinoma	4 (2.29)
Carcinosarcoma	10 (5.71)
Lymphovascular space invasion, *n* (%)	
Positive	39 (22.29%)
Negative	136 (77.71%)
Recurrence, *n* (%)	20 (11.4%)
Locoregional	8 (40%)
Non-locoregional	12 (60%)

**Table 4:** Comparison of MRI parameters between the low-risk and non-low-risk groups.

	Low-Risk Group	Non-Low-Risk Group				*p*-Value
	Low	Intermediate	High-Intermediate	High	Metastatic	
	(*n* = 90)	(*n* = 7)	(*n* = 19)	(*n* = 46)	(*n* = 13)	
Maximum tumor diameter(cm)						<0.001
Median (IQR)	2 (1.3, 3)	3.1 (2.7, 4.7)	3.6 (2.1, 5.2)	4 (2.5, 5.7)	5.1 (2.3, 7.5)	
Growth pattern						0.185
Infiltrative	37 (48.1)	6 (85.7)	8 (42.1)	28 (63.6)	6 (50)	
Expansile	40 (52)	1 (14.3)	11 (57.9)	16 (36.4)	6 (50)	
SI on T2WI						0.802
Hypo or iso	54 (70.1)	5 (71.4)	15 (79)	30 (68.2)	6 (50)	
Hyper	23 (29.9)	2 (28.6)	4 (21.1)	14 (31.8)	6 (50)	
Heterogeneous SI on T2WI						0.193
No	63 (81.8)	6 (85.7)	13 (68.4)	32 (72.7)	9 (75)	
Yes	14 (18.2)	1 (14.3)	6 (31.6)	12 (27.3)	3 (25)	
SI on CET1						0.697
Hypo or iso	66 (85.7)	7 (100)	18 (94.7)	37 (84.1)	10 (83.3)	
Hyper	11 (14.3)	-	1 (5.3)	7 (15.9)	2 (16.7)	
Heterogeneous SI on CET1						0.142
No	58 (75.3)	4 (57.1)	13 (68.4)	29 (65.9)	7 (58.3)	
Yes	19 (24.7)	3 (42.9)	6 (31.6)	15 (34.1)	5 (41.7)	
SI on DWI						0.003
Hypo or iso	22 (29.3)	1 (14.3)	1 (5.9)	5 (11.6)	1 (8.3)	
Hyper	53 (70.7)	6 (85.7)	16 (94.1)	38 (88.4)	11 (91.7)	
Heterogeneous SI on DWI						0.003
No	66 (88)	6 (85.7)	11 (64.7)	31 (72.1)	6 (50)	
Yes	9 (12)	1 (14.3)	6 (35.3)	12 (27.9)	6 (50)	
ADC value						0.419
Median (IQR)	811.2 (719.1, 946.4)	812.6 (761, 895.5)	731.8 (668.8, 822.9)	893.3 (776.7, 960.7)	898.7 (783.8, 1125.1)	
Deep myometrial invasion						<0.001
No	86 (95.6)	3 (42.9)	11 (57.9)	20 (43.5)	7 (53.9)	
Yes	4 (4.4)	4 (57.1)	8 (42.1)	26 (56.5)	6 (46.2)	
Cervical stromal involvement					<0.001	<0.001
No	90 (100)	6 (85.7)	17 (89.5)	36 (78.3)	8 (61.5)	
Yes	-	1 (14.3)	2 (10.5)	10 (21.7)	5 (38.5)	
Extrauterine extension						0.002
No	89 (98.9)	7 (100)	18 (94.7)	40 (87)	9 (69.2)	
Yes	1 (1.1)	-	1 (5.3)	6 (13)	4 (30.8)	
Rectal or bladder invasion						0.113 †
No	90 (100)	7 (100)	18 (94.7)	46 (100)	11 (84.6)	
Yes	-	-	1 (5.3)	-	2 (15.4)	
Abnormal ascites						0.486 †
No	90 (100)	7 (100)	19 (100)	45 (97.8)	13 (100)	
Yes	-	-	-	1 (2.2)	-	
Peritoneal dissemination						0.235 †
No	90 (100)	7 (100)	19 (100)	46 (100)	11 (84.6)	
Yes	-	-	-		2 (15.4)	
Lymphadenopathy						0.003 †
No	90 (100)	7 (100)	18 (94.7)	42 (91.3)	10 (76.9)	
Yes	-	-	1 (5.3)	4 (8.7)	3 (23.1)	

Values are numbers (percentages) for categorical variables and means (SD), median (IQR) others. *p*-values are calculated using a Chi-square test or Fisher’s exact test † for categorical variables and a Wilcoxon rank sum test for continuous variables. SI = signal intensity; T2WI = T2-weighted image; CET1 = contrast-enhanced T1-weighted image; DWI = diffusion-weighted image; ADC = apparent diffusion coefficient.

**Table 5:** Tumor recurrence and FIGO stage discrepancy according to risk group.

	Low-Risk Group	Non-Low-Risk Group				*p*-Value
	Low	Intermediate	High-Intermediate	High	Metastatic	
	(*n* = 90)	(*n* = 7)	(*n* = 19)	(*n* = 46)	(*n* = 13)	
Recurrence						<0.001
No	90 (100)	6 (85.7)	18 (94.7)	35 (76.1)	6 (46.2)	
Yes	-	1 (14.3)	1 (5.3)	11 (23.9)	7 (53.9)	
Stage concordance						<0.001
Concordance	85 (94.4)	5 (71.4)	14 (73.7)	26 (56.5)	6 (46.2)	
Discordance	5 (5.6)	2 (28.6)	5 (26.3)	20 (43.5)	7 (53.9)	

**Table 6:** Comparison of imaging parameters between the p53 wild and p53 mutant groups.

	P53 Wild	p53 Mutant	
	(*n* = 62)	(*n* = 24)	*p*-Value
Maximum tumor diameter (cm)			0.077
Median (IQR)	2.6 (1.5, 3.9)	4 (1.8, 5.3)	
Growth pattern			0.995
Infiltrative	30 (56.6)	13 (56.5)	
Expansile	23 (43.4)	10 (43.5)	
SI on T2WI			0.572
Hypo or iso	38 (71.7)	15 (65.2)	
Hyper	15 (28.3)	8 (34.8)	
Heterogeneous SI on T2WI			0.362
No	42 (79.3)	16 (69.6)	
Yes	11 (20.8)	7 (30.4)	
SI on CET1			0.195 †
Hypo or iso	46 (86.8)	17 (73.9)	
Hyper	7 (13.2)	6 (26.1)	
Heterogeneous SI on CET1			0.552
No	36 (67.9)	14 (60.9)	
Yes	17 (32.1)	9 (39.1)	
SI on DWI			0.669
Hypo or iso	11 (21.6)	6 (26.1)	
Hyper	40 (78.4)	17 (73.9)	
Heterogeneous SI on DWI			>0.999 †
No	41 (80.4)	18 (78.3)	
Yes	10 (19.6)	5 (21.7)	
ADC value			0.182
Median (IQR)	800.7 (712.8, 903.7)	843.1 (777.6, 951.3)	
Deep myometrial invasion			0.752
No	46 (74.2)	17 (70.8)	
Yes	16 (25.8)	7 (29.2)	
Cervical stromal involvement			0.491 †
No	55 (88.7)	20 (83.3)	
Yes	7 (11.3)	4 (16.7)	
Extrauterine extension			0.670 †
No	58 (93.6)	22 (91.7)	
Yes	4 (6.5)	2 (8.3)	
Rectal or bladder invasion			>0.999 †
No	60 (96.8)	24 (100)	
Yes	2 (3.2)	-	
Abnormal ascites			-
No	62 (100)	24 (100)	
Yes	-	-	
Peritoneal dissemination			>0.999 †
No	61 (98.4)	24 (100)	
Yes	1 (1.6)	-	
Lymphadenopathy			>0.999 †
No	60 (96.8)	24 (100)	
Yes	2 (3.2)	-	

Values are numbers (percentages) for categorical variables and means (SD), median (IQR) others. *p*-values are calculated using a Chi-square test or Fisher’s exact test † for categorical variables and a Wilcoxon rank sum test for continuous variables. SI = signal intensity; T2WI = T2-weighted image; CET1 = contrast-enhanced T1-weighted image; DWI = diffusion-weighted image; ADC = apparent diffusion coefficient.

**Table 7:** Comparison of imaging parameters between the MSS and MSI groups.

	MSS	MSI	
	(*n* = 28)	(*n* = 8)	*p*-Value
Maximum tumor diameter (cm)			0.848
Median (IQR)	3.3 (1.7, 4.7)	2.8 (2.3, 4.7)	
Growth pattern			0.175 †
Infiltrative	12 (44.4)	5 (83.3)	
Expansile	15 (55.6)	1 (16.7)	
SI on T2			0.640 †
Hypo or iso	18 (66.7)	5 (83.3)	
Hyper	9 (33.3)	1 (16.7)	
Heterogeneous SI on T2			0.156 †
No	18 (66.7)	6 (100)	
Yes	9 (33.3)	-	
SI on CET1			>0.999 †
Hypo or iso	23 (85.2)	6 (100)	
Hyper	4 (14.8)	-	
Heterogeneous SI on CET1			0.027 †
No	13 (48.2)	6 (100)	
Yes	14 (51.9)	-	
SI on DWI			>0.999 †
Hypo or iso	3 (11.5)	-	
Hyper	23 (88.5)	6 (100)	
Heterogeneous SI on DWI			0.565 †
No	20 (76.9)	6 (100)	
Yes	6 (23.1)	-	
ADC value			0.469
Median (IQR)	822.9 (758.5, 958.9)	784.5 (712.8, 812.6)	
Deep myometrial invasion			0.384 †
No	22 (78.6)	5 (62.5)	
Yes	6 (21.4)	3 (37.5)	
Cervical stromal involvement			>0.999 †
No	24 (85.7)	7 (87.5)	
Yes	4 (14.3)	1 (12.5)	
Extrauterine extension			0.400 †
No	27 (96.4)	7 (87.5)	
Yes	1 (3.6)	1 (12.5)	
Rectal or bladder invasion			-
No	28 (100)	8 (100)	
Yes	-	-	
Abnormal ascites			-
No	28 (100)	8 (100)	
Yes	-	-	
Peritoneal dissemination			-
No	28 (100)	8 (100)	
Yes	-	-	
Lymphadenopathy			-
No	28 (100)	8 (100)	
Yes	-	-	

Values are numbers (percentages) for categorical variables and means (SD), median (IQR) others. *p*-values are calculated using a Chi-square test or Fisher’s exact test † for categorical variables and a Wilcoxon rank sum test for continuous variables. MSS = microsatellite stable; MSI = microsatellite instability; SI = signal intensity; T2WI = T2-weighted image; CET1 = contrast-enhanced T1-weighted image; DWI = diffusion-weighted image; ADC = apparent diffusion coefficient.

## Data Availability

The datasets generated and/or analyzed during the current study are available from the corresponding author upon reasonable request.
